# Effects of sodium-glucose co-transporter 2 (SGLT2) inhibition on renal function and albuminuria in patients with type 2 diabetes: a systematic review and meta-analysis

**DOI:** 10.7717/peerj.3405

**Published:** 2017-06-27

**Authors:** Lubin Xu, Yang Li, Jiaxin Lang, Peng Xia, Xinyu Zhao, Li Wang, Yang Yu, Limeng Chen

**Affiliations:** 1 Department of Nephrology, Peking Union Medical College Hospital, Chinese Academy of Medical Sciences and Peking Union Medical College, Beijing, China; 2 Department of Epidemiology and Biostatistics, Institute of Basic Medical Sciences, Chinese Academy of Medical Sciences, and School of Basic Medicine, Peking Union Medical College, Beijing, China

**Keywords:** SGLT2 inhibitor, Glomerular filtration rate, Albuminuria, Diabetic nephropathy, Meta-analysis

## Abstract

**Aim:**

To evaluate the effects of sodium-glucose co-transporter 2 (SGLT2) inhibition on renal function and albuminuria in patients with type 2 diabetes.

**Methods:**

We conducted systematic searches of PubMed, Embase and Cochrane Central Register of Controlled Trials up to June 2016 and included randomized controlled trials of SGLT2 inhibitors in adult type 2 diabetic patients reporting estimated glomerular filtration rate (eGFR) and/or urine albumin/creatinine ratio (ACR) changes. Data were synthesized using the random-effects model.

**Results:**

Forty-seven studies with 22,843 participants were included. SGLT2 inhibition was not associated with a significant change in eGFR in general (weighted mean difference (WMD), −0.33 ml/min per 1.73 m^2^, 95% CI [−0.90 to 0.23]) or in patients with chronic kidney disease (CKD) (WMD −0.78 ml/min per 1.73 m^2^, 95% CI [−2.52 to 0.97]). SGLT2 inhibition was associated with eGFR reduction in short-term trials (WMD −0.98 ml/min per 1.73 m^2^, 95% CI [−1.42 to −0.54]), and with eGFR preservation in long-term trials (WMD 2.01 ml/min per 1.73 m^2^, 95% CI [0.86 to 3.16]). Urine ACR reduction after SGLT2 inhibition was not statistically significant in type 2 diabetic patients in general (WMD −7.24 mg/g, 95% CI [−15.54 to 1.06]), but was significant in patients with CKD (WMD −107.35 mg/g, 95% CI [−192.53 to −22.18]).

**Conclusions:**

SGLT2 inhibition was not associated with significant changes in eGFR in patients with type 2 diabetes, likely resulting from a mixture of an initial reduction of eGFR and long-term renal function preservation. SGLT2 inhibition was associated with statistically significant albuminuria reduction in type 2 diabetic patients with CKD.

## Introduction

With increasing incidence and prevalence of diabetes mellitus, diabetic nephropathy has become the leading cause of end-stage renal disease (ESRD), accounting for 50% of cases in the developed world (Tuttle et al., 2014; Wild et al., 2004). Current management of diabetic nephropathy includes avoidance of nephrotoxic agents, prevention of infections, glycemic control, and blood pressure control, with emphasis on the use of renin-angiotensin-aldosterone system (RAAS) inhibitors. However, these strategies only provide partial renoprotection against progression of diabetic nephropathy (Bilous et al., 2009; Lewis et al., 1993; Lewis et al., 2001; Mauer et al., 2009). Thus, additional therapeutic interventions for the prevention and treatment of diabetic nephropathy are needed.

Sodium-glucose co-transporter 2 (SGLT2) inhibitors, including canagliflozin, dapagliflozin, empagliflozin, ipragliflozin and tofogliflozin, are a new class of antihyperglycemic drugs that lower blood glucose by blocking glucose reabsorption via SGLT2 at the proximal renal tubule. SGLT2 inhibitors are gaining popularity due to their various beneficial effects. In addition to glycemic control, SGLT2 inhibitors lower blood pressure, control body weight, and reduce cardiovascular mortality in type 2 diabetic patients with high cardiovascular risk (Baker et al., 2014; Matthaei et al., 2015; Tikkanen et al., 2015; Wilding et al., 2015; Zinman et al., 2015b).

SGLT2 inhibition also has profound effects on renal hemodynamics. The tubular hypothesis implicates that impaired tubuloglomerular feedback (TGF) due to upregulation of SGLT2 plays a central role in hyperfiltration in diabetic patients, leading to albuminuria and decline in renal function (Skrtic & Cherney, 2015). While RAAS activation mainly leads to vasoconstriction of efferent arterioles (Sochett et al., 2006), impairment of TGF mediates hyperfiltration via vasodilation of afferent arterioles. SGLT2 inhibitors block glucose and sodium reabsorption at the proximal tubule, increase sodium delivery to the macula densa, and consequently restore impaired TGF. Thus, it is postulated that SGLT2 inhibition alleviates glomerular hyperfiltration in the early stages of diabetic nephropathy, reduces albuminuria, and slows the decline of renal function in the long term. These effects have been observed in micropuncture studies conducted in rats and a proof-of-concept study of type 1 diabetic patients with hyperfiltration (Cherney et al., 2014; Skrtic & Cherney, 2015; Thomson et al., 2012).

A number of clinical trials have reported kidney-related outcomes after SGLT2 inhibitor use (Bailey et al., 2015; Barnett et al., 2014; Wanner et al., 2016; Yale et al., 2013). In the EMPA-REG OUTCOME trial, empagliflozin reduced incident or worsening nephropathy and slowed decline of renal function in type 2 diabetic patients at high cardiovascular risk (Wanner et al., 2016). A previous meta-analysis (Liu et al., 2015) up to December 2014 has concluded that SGLT2 inhibition does not have a significant effect on the estimated glomerular filtration rate (eGFR). However, there has been no up-to-date systematic review examining whether SGLT2 inhibitors attenuate hyperfiltration in acute settings and in the early stages of diabetic nephropathy, whether SGLT2 inhibitors preserve GFR in the long term and for patients with more advanced nephropathy, or whether SGLT2 inhibitors reduce albuminuria. Thus, we conducted this meta-analysis of randomized controlled trials (RCTs) to thoroughly characterize the effects of SGLT2 inhibitors on eGFR and albuminuria compared with placebo or other antidiabetic treatments in patients with type 2 diabetes.

## Materials and Methods

This review conforms to the standard guidelines and was written according to the Preferred Reporting Items for Systematic Reviews and Meta-Analyses (PRISMA) statement (Moher et al., 2009).

### Search strategy

We conducted a systematic search of PubMed, Embase and Cochrane Central Register of Controlled Trials (CENTRAL) databases through June 19th 2016. The search strategy is provided in Item S3; we used medical subject headings, as well as free-text search terms, including SGLT2 inhibitors, canagliflozin, dapagliflozin, empagliflozin, atigliflozin, ‘bi 44847’, ertugliflozin, ipragliflozin, luseogliflozin, remogliflozin, sergliflozin, sotagliflozin and tofogliflozin. We also conducted a manual search of references of existing reviews in this field to identify additional relevant studies.

### Study selection

Two reviewers (LX and YL) independently screened the search results and retrieved relevant studies for further evaluation. The retrieved full-text articles were examined by two reviewers (LX and YL) in parallel for inclusion according to predetermined criteria. We included RCTs conducted on adult type 2 diabetic patients that compared SGLT2 inhibitors with either placebo or other antidiabetic drugs and reported changes in eGFR and/or urine albumin/creatinine ratio (ACR). Only manuscripts published in English were included. For studies reporting renal outcomes in forms other than eGFR and urine ACR (i.e., serum creatinine, urine protein excretion, etc.), an e-mail was sent to the corresponding author requesting further data. For multiple publications from the same study, only the first publication reporting renal outcomes was included. Disagreement was resolved by discussion and/or consultation with a third reviewer (PX).

### Data extraction and validity assessment

Two reviewers (LX and JL) independently used a standard data extraction tool to record the following properties of each study: the study characteristics (author, year, study design, method of randomization, duration of follow-up, and number of dropouts), participant characteristics (sample size, age, sex, duration of diabetes, baseline HbA1C level, baseline blood pressure, eGFR and urine ACR), therapeutic intervention (type of SGLT2 inhibitor, dose, frequency and duration of treatment), concomitant therapies (concomitant antidiabetic therapy and RAAS inhibitors), comparison groups (placebo-controlled or active-controlled), outcomes of interest (means and standard deviations (SDs) of changes in eGFR and urine ACR in treatment and control groups), whether outcomes of chronic kidney disease (CKD) subjects (defined as eGFR < 60 ml/min per 1.73 m^2^ or microalbuminuria or macroalbuminuria) were reported, and the funding source. The program g3data (www.frantz.fi/software/g3data.php) was used to extract relevant data that were reported in figures but not in the text.

Study quality was evaluated by two authors (LX and YL) independently using the ‘Risk of bias’ assessment tool from the *Cochrane Handbook for Systematic Reviews of Interventions*, version 5.1 (2011). The domains of assessment included random sequence generation, allocation concealment, blinding of participants and personnel, blinding of outcome assessment, incomplete outcome data, selective reporting and other bias.

### Statistical analysis

Outcome measures for each trial were mean differences of changes (calculated as (end of trial value for treatment group—baseline value for treatment group)—(end of trial value for control group—baseline value for control group)) in eGFR and urine ACR. For studies in which SD was not directly reported, SD was calculated from SE (standard error) or 95% confidence intervals (CI), or imputed as recommended in the *Cochrane Handbook for Systematic Reviews of Interventions*, version 5.1 using a correlation coefficient of 0.8 (with the formula provided in Item S4) (Higgins & Green, 2011). For studies with more than one SGLT2 treatment arm, these groups were combined to create a single treatment arm (with the formulae provided in Item S5) (Higgins & Green, 2011). The inverse variance method was used to estimate the pooled weighted mean differences (WMDs) in eGFR and urine ACR. As clinical and statistical heterogeneity were anticipated, we decided *a priori* to use the random-effects model in our data synthesis.

Statistical heterogeneity was quantified using the Cochrane *Q* test and *I*^2^ statistic. Subgroup analysis was planned for the type of SGLT2 inhibitor, placebo or active control, concomitant use of RAAS inhibitors, trial duration, mean baseline age, mean duration of diabetes, mean baseline HbA1C level, whether the study population were CKD patients (eGFR < 60 ml/min per 1.73 m^2^ or microalbuminuria or macroalbuminuria), and whether the study population had hyperfiltration (eGFR ≥ 125 ml/min per 1.73 m^2^) (Dahlquist, Stattin & Rudberg, 2001). Test for subgroup differences were carried out using RevMan 5.3 (The Nordic Cochrane Centre, The Cochrane Collaboration).

The possibility of publication bias was assessed using funnel plots and Egger’s test. Sensitivity analysis excluding trials with relatively high risk (defined as ≥1 item with high risk or ≥2 items with unclear risk in the ‘Risk of Bias’ assessment tool) was performed.

Statistical analyses were performed using the Stata 12.0 software package (StataCorp, LP, College Station, TX) and RevMan 5.3 (The Nordic Cochrane Centre, The Cochrane Collaboration). Statistical significance was set at *p* < 0.05 for all analyses.

## Results

### Study selection and characteristics

**Figure 1:**
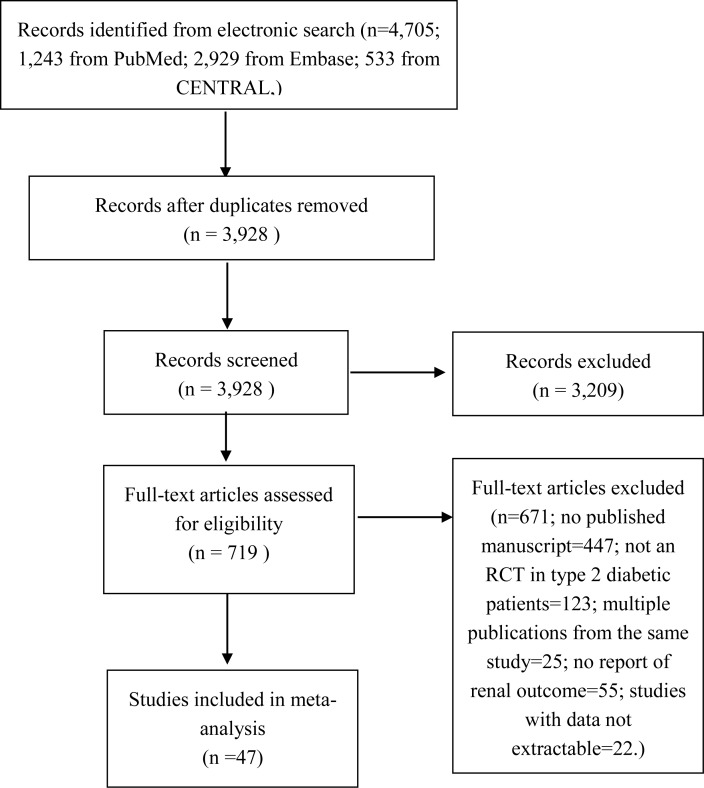
Identification process for eligible studies. Abbreviations: CENTRAL, Cochrane Central Register of Controlled Trials; RCT, randomized controlled trial.

The results of the literature search and study selection are shown in Fig. 1. Details of the study selection process can be found in Item S6. This search process led to inclusion of 47 studies (Bailey et al., 2015; Barnett et al., 2014; Bode et al., 2013; Bolinder et al., 2012; Cefalu et al., 2013; DeFronzo et al., 2015; Fonseca et al., 2013; Forst et al., 2014; Haring et al., 2014; Haring et al., 2013; Inagaki et al., 2014; Ji et al., 2015; Ji et al., 2014; Kadowaki et al., 2014; Kaku et al., 2014; Kashiwagi et al., 2015a; Kashiwagi et al., 2015b; Kashiwagi et al., 2015c; Kashiwagi et al., 2015d; Kohan et al., 2014; Kovacs et al., 2014; Lambers Heerspink et al., 2013; Lavalle-Gonzalez et al., 2013; Lewin et al., 2015; Lu et al., 2016; Nauck et al., 2011; Nishimura et al., 2015; Qiu, Capuano & Meininger, 2014; Ridderstrale et al., 2014; Rodbard et al., 2016; Roden et al., 2013; Rosenstock et al., 2016; Rosenstock et al., 2014; Rosenstock et al., 2015; Ross et al., 2015; Schernthaner et al., 2013; Schumm-Draeger et al., 2015; Sha et al., 2014; Strojek et al., 2011; Tikkanen et al., 2015; Wanner et al., 2016; Weber et al., 2016; Wilding et al., 2013a; Wilding et al., 2013b; Wilding et al., 2009; Wilding et al., 2012; Yale et al., 2013) with 22,843 participants in our meta-analysis.

**Table 1:** Characteristics of included studies.

Study	Dose	Control group	Duration of follow-up (weeks)	Sample size	Mean age (years)	Mean duration of diabetes (years)	Mean baseline HbA1C (%)	Mean baseline blood pressure (mmHg)	Mean baseline eGFR (ml/min/ 1.73 m^2^)	Mean baseline urine ACR (mg/g)	Reported outcomes of CKD patients	Outcomes reported
CANAGLIFLOZIN												
Bode et al. (2013)	100 mg, 300 mg	Placebo	26	584	63.6	11.7	7.7	131.0/75.7	77.5	N.R.	No	eGFR
Cefalu et al. (2013)	100 mg, 300 mg	Glimepiride	52	1,038	56.2	6.6	7.8	129.8/79.0	N.R.	29.1	No	eGFR, uACR
Lavalle-Gonzalez et al. (2013)	100 mg, 300 mg	Sitagliptin	52	873	55.5	6.9	7.9	128.2/77.7	89.7	N.R.	No	eGFR
Schernthaner et al. (2013)	300 mg	Sitagliptin	52	460	56.7	9.6	8.1	130.7/78.9	88.9	N.R.	No	eGFR
Wilding et al. (2013a) IJCP	100 mg, 300 mg	Placebo	52	306	56.8	9.6	8.1	130.4/78.7	90.3	N.R.	No	eGFR
Yale et al. (2013)	100 mg, 300 mg	Placebo	26	211	68.5	16.3	8.0	134.9/74.4	39.4	30.0 (median)	Yes	eGFR, uACR
Forst et al. (2014)	100 mg, 300 mg	Placebo (26 weeks) + sitagliptin (26 weeks)	52	261	57.4	10.5	7.9	127.1/76.4	86.4	N.R.	No	eGFR
Inagaki et al. (2014)	100 mg, 200 mg	Placebo	24	240	58.0	5.4	8.0	127.9/77.8	84.4	N.R.	No	uACR
Qiu, Capuano & Meininger (2014)	50 mg bid, 150 mg bid	Placebo	18	239	57.4	7.0	7.6	129.3/78.1	85.9	N.R.	No	eGFR
Sha et al. (2014)	300 mg	Placebo	12	35	62.8	8.5	7.7	132.9/80.0	97.3	N.R.	No	eGFR
Ji et al. (2015)	100 mg, 300 mg	Placebo	18	636	56.2	6.7	8.0	129.5/77.3	94.0	N.R.	No	eGFR
Rodbard et al. (2016)	100 mg or 300 mg (titrated)^a^	Placebo	26	171	57.4	9.9	8.5	N.R.	90.5	N.R.	No	eGFR
Rosenstock et al. (2016)	100 mg, 300 mg	Placebo	26	618	54.9	3.2	8.8	128.6/78.3	87.0	N.R.	No	eGFR
DAPAGLIFLOZIN												
Wilding et al. (2009)	10 mg, 20 mg	Placebo	12	68	56.7	12.3	8.4	128.8/77.4	87.6	N.R.	No	eGFR
Strojek et al. (2011)	2.5 mg, 5 mg, 10 mg	Placebo	24	596	59.8	7.4	8.1	N.R.	76.7	N.R.	No	eGFR
Nauck et al. (2011)	2.5 mg–10 mg (titrated)^b^	Glipizide	52	814	58.5	6.5	7.7	133.3/80.6	90.1	58.0	No	eGFR, uACR
Bolinder et al. (2012)	10 mg	Placebo	24	167	60.7	5.8	7.2	134.6/80.5	84.3	44.3	No	eGFR, uACR
Wilding et al. (2012)	2.5 mg, 5 mg, 10 mg	Placebo	48	658	59.3	13.6	8.5	138.5/80.1	78.4	75.2	No	eGFR, uACR
Lambers Heerspink et al. (2013)	10 mg	Placebo	12	48	55.9	6.5	7.6	136.41/82.0	N.R.	N.R.	No	eGFR
Ji et al. (2014)	5 mg, 10 mg	Placebo	24	338	51.4	1.4	8.3	123.6/77.8	92.5	N.R.	No	eGFR
Kohan et al. (2014)	5 mg, 10 mg	Placebo	104	132	67.0	16.9	8.4	132.1/73.3	44.6	N.R.	Yes	eGFR, uACR
Schumm-Draeger et al. (2015)	2.5 mg bid, 5 mg bid, 10 mg qd	Placebo	16	400	57.7	5.2	7.8	132.0/80.7	86.3	N.R.	No	eGFR
Bailey et al. (2015)	2.5 mg, 5 mg, 10 mg	Placebo 24 weeks + 500 mg metformin	102	274	52.2	1.9	7.9	125.9/80.5	85.1	23.5	No	eGFR, uACR
Weber et al. (2016)	10 mg	Placebo	12	449	56.5	7.5	8.1	151.1/91.3	85.9	143.7	No	eGFR, uACR
EMPAGLIFLOZIN												
Haring et al. (2013)	10 mg, 25 mg	Placebo	24	666	57.1	N.R.	8.1	128.9/78.6	87.2	N.R.	No	eGFR
Roden et al. (2013)	10 mg, 25 mg	Placebo	24	676	54.7	N.R.	7.9	131.1/78.8	N.R.	N.R.	No	eGFR
Barnett et al. (2014)	10 mg, 25 mg	Placebo	52	637	64.1	N.R.	8.0	135.3/76.9 (CKD2)	71.6 (CKD2)	155.0 (CKD2)	Yes	eGFR, uACR
									133.7/76.7 (CKD3)	44.9 (CKD3)	362.5 (CKD3)	
									145.6/77.6 (CKD4)	23.2 (CKD4)	1387.4 (CKD4)	
Haring et al. (2014)	10 mg, 25 mg	Placebo	24	637	55.7	N.R.	7.9	129.4/78.7	89.0	N.R.	No	eGFR
Kovacs et al. (2014)	10 mg, 25 mg	Placebo	24	498	54.5	N.R.	8.1	126.1/76.9	85.7	N.R.	No	eGFR
Rosenstock et al. (2014)	10 mg, 25 mg	Placebo	52	563	56.7	N.R.	8.3	126.2/78.2	N.R.	N.R.	No	eGFR
Ridderstrale et al. (2014)	25 mg	Glimepiride	104	1500	56.0	N.R.	7.9	133.5/79.5	88.0	40.2^c^	Yes	eGFR, uACR
Kadowaki et al. (2014)	5 mg, 10 mg, 25 mg, 50 mg	Placebo	12	547	57.5	N.R.	8.0	129.2/78.7	85.7	N.R.	No	eGFR
DeFronzo et al. (2015)	10 mg, 25 mg	Linagliptin	52	313	55.9	N.R.	8.0	129.8/79.3	90.5	52.2	No	eGFR, uACR
Lewin et al. (2015)	10 mg, 25 mg	Linagliptin	52	340	54.6	N.R.	8.0	128.5/78.8	88.8	36.8	No	eGFR, uACR
Tikkanen et al. (2015)	10 mg, 25 mg	Placebo	12	723	60.2	N.R.	7.9	142.1/83.9	84.0	N.R.	No	eGFR
Nishimura et al. (2015)	10 mg, 25 mg	Placebo	4	60	62.7	N.R.	7.9	120.9/72.4	80.0	N.R.	No	eGFR
Rosenstock et al. (2015)	10 mg, 25 mg	Placebo	78	364	58.8	N.R.	8.2	133.0/78.3	84.0	N.R.	No	eGFR
Ross et al. (2015)	12.5 mg bid, 25 mg qd, 5 mg bid, 10 mg qd	Placebo	16	965	58.2	N.R.	7.8	131.3/78.6	89.2	N.R.	No	eGFR
Wanner et al. (2016)	10 mg, 25 mg	Placebo	156(median)	3064	63.1	N.R.	8.07	135.5/76.7	74.1	N.R.	Yes	eGFR
IPRAGLIFLOZIN												
Wilding et al. (2013b) DOM	12.5 mg, 50 mg, 150 mg, 300 mg	Placebo	12	304	57.4	5.9	7.8	N.R.	N.R.	N.R.	No	eGFR
Fonseca et al. (2013)	12.5 mg, 50 mg, 150 mg, 300 mg	Placebo	12	304	53.7	4.6	7.9	N.R.	N.R.	N.R.	No	eGFR
Kashiwagi et al. (2015a) DI EMIT	50 mg	Placebo	24	240	59.7	10.5	8.4	130.0/76.6	84.7	50.8	No	eGFR, uACR
Kashiwagi et al. (2015b) DI BRIGHTEN	50 mg	Placebo	16	129	59.4	6.7	8.3	130.0/128.2	87.8	N.R.	No	eGFR
Kashiwagi et al. (2015c) DI SPOTLIGHT	50 mg	Placebo	24	151	56.2	6.8	9.3	130.4/77.9	91.0	39.3	No	eGFR, uACR
Kashiwagi et al. (2015d) DOM LANTERN	50 mg	Placebo	24	164	64.4	9.5	7.5	133.3/77.3	60.9	148.2	Yes	eGFR, uACR
Lu et al. (2016)	50 mg	Placebo	24	170	53.7	6.2	7.7	N.R.	149.2	N.R.	No	eGFR, uACR
TOFOGLIFLOZIN												
Kaku et al. (2014)	10 mg, 20 mg, 40 mg	Placebo	24	212	57.3	6.4	8.4	129.2/78.3	85.4	N.R.	No	eGFR

**Notes.**

a After six weeks, the canagliflozin dose was increased from 100 to 300 mg (or from placebo to matching placebo) if all of the following criteria were met: baseline eGFR ≥ 70 ml/min/1.73 m^2^; fasting self-monitored blood glucose ≥ 5.6 mmol/l (100 mg/dl); and no volume depletion-related adverse events within two weeks before dose increase.

b From week 0 to week 18 (titration period), patients received an initial dose of dapagliflozin of 2.5 mg, which was up-titrated for patients with fasting blood glucose ≥ 110 mg/dl (6.1 mmol/l) until the maximum dose of 10 mg was reached. From week 19 to week 52 (maintenance period), the dose was no longer up-titrated but could be down-titrated in the event of recurrent hypoglycemia.

c Mean baseline urine ACRs: normoalbuminuric group, 9.55 mg/g; microalbuminuric group, 86.3 mg/g; and macroalbuminuric group, 728.9 mg/g.

Abbreviations N.R. not reported uACR urine albumin/creatinine ratio IJCP International Journal of Clinical Practice DOM Diabetes, Obesity and Metabolism DI Diabetology International

EMIT, BRIGHTEN, SPOTLIGHT, and LANTERN are names of randomized controlled trials.

The characteristics of the included studies are shown in Table 1. Of the 47 studies, 46 studies with 22,603 participants reported changes in eGFR, and 17 studies with 7,285 participants reported changes in urine ACR. Five SGLT2 inhibitors, including dapagliflozin, canagliflozin, empagliflozin, ipragliflozin and tofogliflozin, were assessed. A total of 38 studies were placebo-controlled, and 9 were controlled by other antidiabetic medications, including metformin, glimepiride, glipizide, linagliptin, and sitagliptin. The trial durations ranged from 4 weeks to 156 weeks. Six studies reported outcomes of CKD subjects. In other studies, the mean baseline eGFR ranged from 76.7 to 149.2 ml/min per 1.73 m^2^, and the mean baseline urine ACR ranged from 6.7 to 143.7 mg/g. None of the studies reported outcomes in a group of patients with hyperfiltration. Collection of data on concomitant use of RAAS inhibitors was planned *a priori*; however, this was impeded as only six studies reported the number of patients using RAAS inhibitors (Barnett et al., 2014; Bode et al., 2013; Lambers Heerspink et al., 2013; Sha et al., 2014; Wanner et al., 2016; Weber et al., 2016), among which only one study reported outcomes with stratification according to RAAS inhibitor usage (Weber et al., 2016).

Quality assessment (Figs. S1 and S2) revealed that 32 studies described random sequence generation, and that 34 described allocation concealment. Twenty-seven studies described blinding of participants and personnel. All 47 studies had a low risk of detection or reporting bias, and 31 studies had a low risk of attrition bias.

### Assessment of publication bias

Visual inspection revealed some asymmetry in the funnel plots for both eGFR (Fig. S3) and urine ACR (Fig. S4). Egger’s regression confirmed statistical significance of publication bias for urine ACR (*p* = 0.002), but not eGFR (*p* = 0.057).

### Effects of SGLT2 inhibitors on eGFR

**Figure 2:**
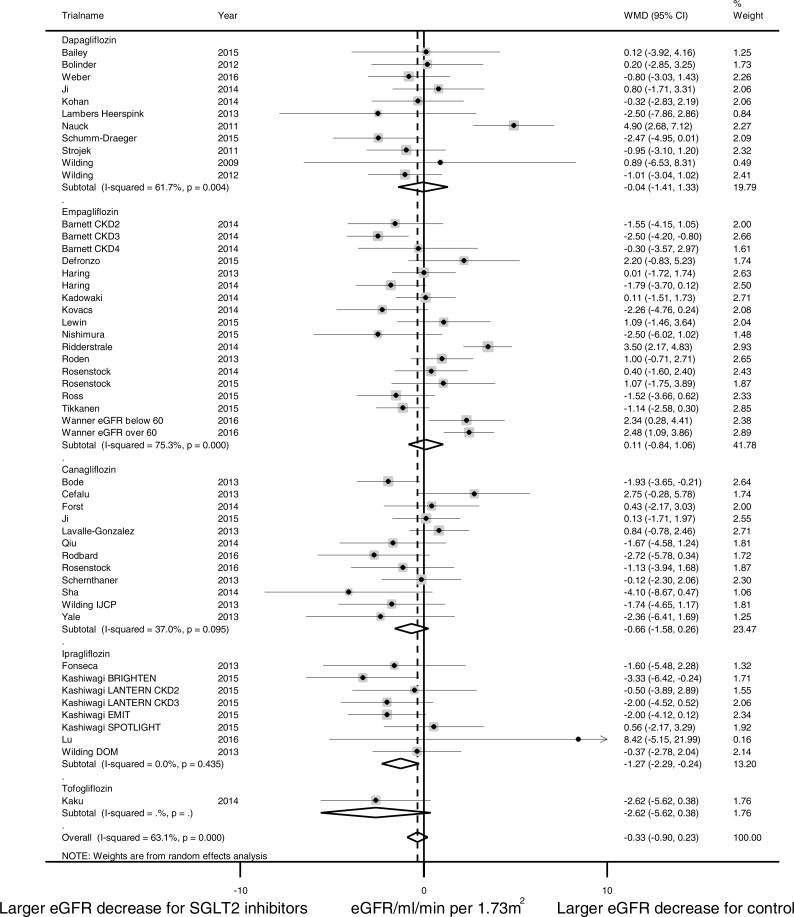
Effect of SGLT2 inhibition on eGFR. The black dots represent mean differences of changes in eGFR, calculated as ((end of trial eGFR for SGLT2 inhibition–baseline eGFR for SGLT2 inhibition)–(end of trial eGFR for control—baseline eGFR for control)). The gray squares represent weights calculated using the random-effects model. The horizontal lines represent 95% confidence intervals (CIs). The hollow diamonds represent pooled mean differences and their 95% CIs. Negative values indicate that SGLT2 inhibitors had larger eGFR decrease than control. eGFR in ml/min per 1.73 m^2^.

In pooled analysis of 46 studies reporting eGFR changes, no significant difference was observed between the SGLT2 treatment group and control group (calculated as ((end of trial eGFR for SGLT2 inhibition group—baseline eGFR for SGLT2 inhibition group)—(end of trial eGFR for control group—baseline eGFR for control group))) (WMD −0.33 ml/min per 1.73 m^2^, (95% CI [−0.90 to 0.23]); Fig. 2). Substantial heterogeneity was detected across studies (*I*^2^ = 63.1%, *p* < 0.001).

**Figure 3:**
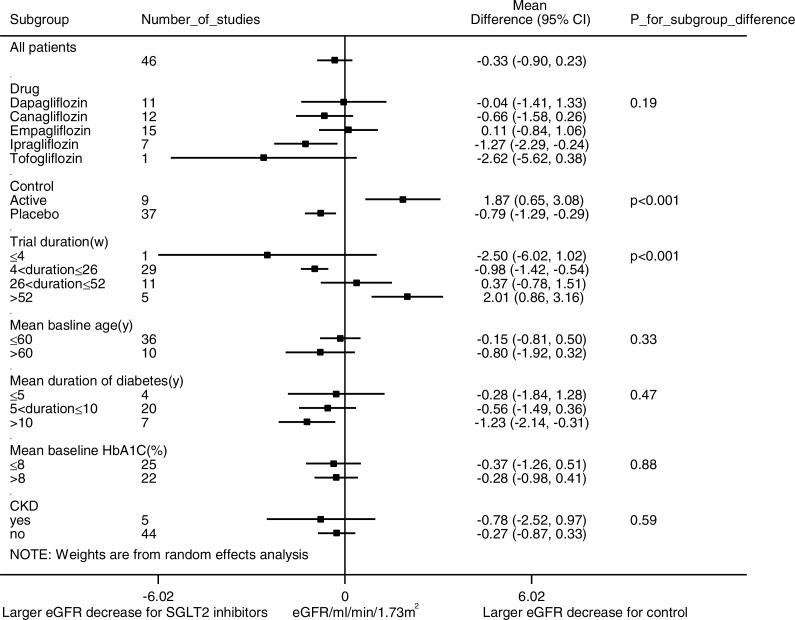
Subgroup analysis of the effect of SGLT2 inhibition on eGFR. Pre-specified subgroup analyses were performed to address sources of heterogeneity. Weighted mean differences for eGFR are represented by small squares. The horizontal lines show 95% confidence intervals. The *P* values for subgroup differences are listed. Negative values indicate that the eGFR decrease was larger in the SGLT2 inhibition group compared with the control group.

Pre-specified subgroup analyses were conducted to identify possible sources of heterogeneity (Fig. 3). Analysis of trial duration revealed that in the trials with a duration of 4–26 weeks, SGLT2 inhibition was associated with a larger eGFR reduction than control (WMD -0.98 ml/min per 1.73 m^2^, (95% CI [−1.42 to −0.54]), *I*^2^ = 0.9%, 29 studies with 10,946 patients); while in trials that lasted longer than 52 weeks, SGLT2 inhibition was associated with slower eGFR decline than control (WMD 2.01 ml/min per 1.73 m^2^, (95% CI [0.86 to 3.16]), *I*^2^ = 46.0%, 5 studies with 5,334 patients). SGLT2 inhibitors were observed with a larger eGFR reduction than placebo and a smaller eGFR reduction than active control. No significant eGFR difference was observed between the SGLT2 inhibitor group and control group in patients with CKD (WMD −0.78 ml/min per 1.73 m^2^, (95% CI [−2.52 to 0.97]), *I*^2^ = 65.0%, 5 studies with 1,574 patients). No significant subgroup differences were observed in subgroup analysis for the type of SGLT2 inhibitor, the mean duration of diabetes, or the mean baseline HbA1C level. Subgroup analyses for patients with hyperfiltration and RAAS inhibitor use had been planned but were hampered by lack of such stratification in the published data.

### Effects of SGLT2 inhibitors on urine ACR

**Figure 4:**
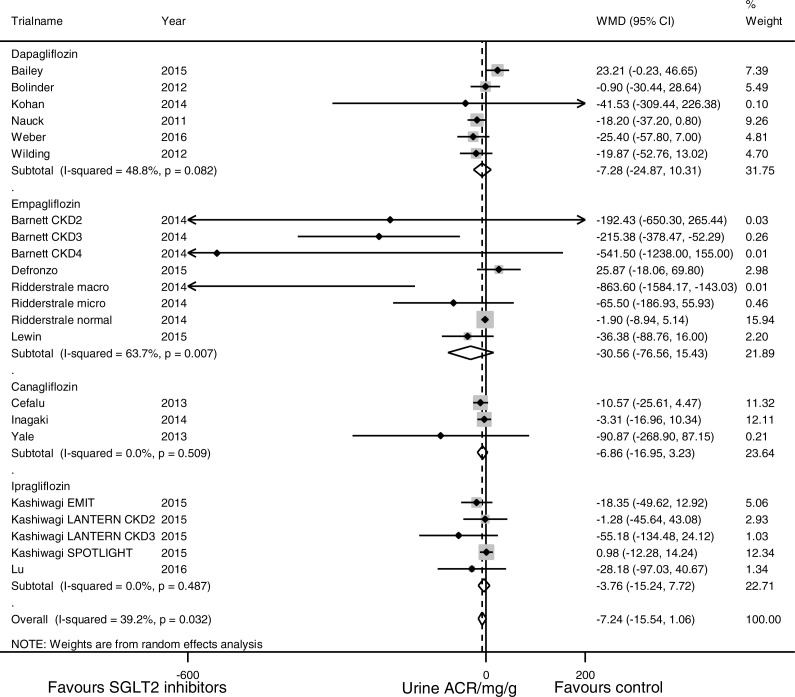
Effect of SGLT2 inhibition on urine albumin/creatinine ratio (ACR). The black dots represent mean differences of changes in urine ACR, calculated as ((end of trial urine ACR for SGLT2 inhibition–baseline urine ACR for SGLT2 inhibition)–(end of trial urine ACR for control—baseline urine ACR for control)). The gray squares represent weights calculated using the random-effects model. The horizontal lines represent 95% confidence intervals (CIs). The hollow diamonds represent pooled mean differences and their 95% CIs. Negative values indicate that the SGLT2 inhibition group had less albuminuria than control. Urine ACR in mg/g.

In pooled analysis of 17 studies evaluating the urine ACR, SGLT2 inhibition was not associated with statistically significant albuminuria reduction (WMD −7.24 mg/g, (95% CI [−15.54 to 1.06]), Fig. 4). Substantial heterogeneity was observed across studies (*I*^2^ = 39.2%, *p* = 0.03).

**Figure 5:**
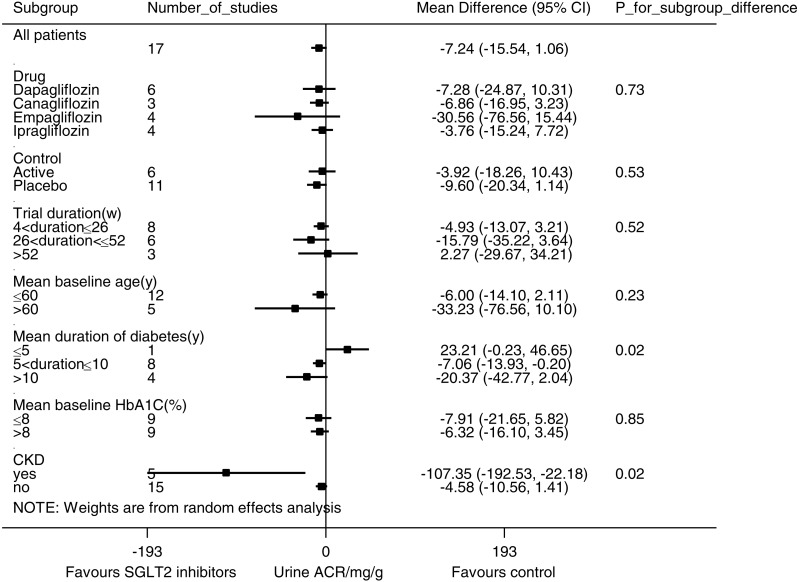
Subgroup analysis of the effect of SGLT2 inhibition on urine albumin/creatinine ratio (ACR). Pre-specified subgroup analyses were performed to address sources of heterogeneity. Weighted mean differences for urine ACR are represented by small squares. The horizontal lines show 95% confidence intervals. The *P* values for subgroup differences are listed. Negative values indicate that the SGLT2 inhibition group had less albuminuria than the control group.

Subgroup analyses (Fig. 5) suggested that SGLT2 inhibition was associated with a significant urine ACR reduction in the participants with CKD (WMD −107.35 mg/g, (95% CI [−192.53 to −22.18]), *I*^2^ = 35.7%, 5 studies with 1,063 participants). Stratification according to the duration of diabetes mellitus revealed a trend of enhanced albuminuria reduction in the patients with a longer duration of diabetes mellitus (for the patients with a history of ≤5 years, WMD 23.21 mg/g, (95% CI [−0.23 to 46.65]), for the patients with a history of 5–10 years, WMD −7.06 mg/g, (95% CI [−13.93 to −0.20]), and for the patients with a history of >10 years, WMD −20.37 mg/g, (95% CI [−42.77 to 2.04]), *p* = 0.02 for subgroup difference). Subgroup analyses for the different SGLT2 inhibitors, use of placebo vs. active control, trial duration, mean baseline age and mean baseline HbA1C level did not reveal any significant subgroup difference.

### Sensitivity analysis

Similar results were observed when analyses were limited to trials with relatively low risk (defined as no item with high risk and no more than 1 item with unclear risk) for eGFR (WMD −0.06 ml/min per 1.73 m^2^, (95% CI [−0.90 to 0.78]), *I*^2^ = 73.0%, 24 trials with 14,535 participants) and urine ACR (WMD −7.25 mg/g, (95% CI [−17.25 to 2.76]), *I*^2^ = 48.0%, 12 trials with 5,308 participants).

## Discussion

In this systematic review and meta-analysis, we identified no significant effect of SGLT2 inhibition on eGFR either in type 2 diabetic patients in general or in type 2 diabetic patients with CKD. Subgroup analysis suggested dipping of eGFR in shorter trials and preservation of eGFR in trials of longer duration. Urine ACR reduction after SGLT2 inhibition was not statistically significant in type 2 diabetic patients in general, but was significant in patients with CKD.

SGLT2 inhibitors can exert their effects on the diabetic kidney through several different mechanisms. First, SGLT2 inhibitors can reverse hyperfiltration and attenuate albuminuria by restoring impaired TGF (Cherney et al., 2014; Vallon et al., 2014). In patients with diabetes, upregulation of SGLT2 increases reabsorption of sodium and glucose along the proximal tubules (Rahmoune et al., 2005), attenuates macula densa-mediated vasoconstriction of afferent arterioles, and results in an increased GFR. SGLT2 inhibition is thought to restore impaired TGF and to reverse hyperfiltration (Skrtic & Cherney, 2015). Second, SGLT2 inhibitors have been shown to alleviate inflammation and to protect the kidney by reducing glucose trafficking through proximal tubule cells (Panchapakesan et al., 2013). Third, SGLT2 inhibition can protect the kidney through systematic changes, including enhanced glycemic control, osmotic diuresis, natriuresis (Rajasekeran, Lytvyn & Cherney, 2016), blood pressure lowering (Baker et al., 2014; Weber et al., 2016), and weight loss (Zinman et al., 2015a).

In our meta-analysis, we identified no statistically significant impact of SGLT2 inhibitors on eGFR in type 2 diabetic patients overall, in line with a previous meta-analysis (Liu et al., 2015). However, this might result from a mixture of initial eGFR dipping and long-term eGFR preservation. We noticed a pattern of eGFR reduction in the short-term studies and eGFR preservation in the longer-term studies, as has been reported in several clinical trials (Cefalu et al., 2013; Kohan et al., 2014; Lambers Heerspink et al., 2013; Strojek et al., 2011; Wanner et al., 2016; Yale et al., 2013), including the EMPA-REG OUTCOME study (Wanner et al., 2016), and pooled analyses (Kohan et al., 2016; Yamout et al., 2014). This pattern, as well as the reversibility of eGFR after drug discontinuation (Barnett et al., 2014; Wanner et al., 2016), suggests that initial reduction of eGFR is probably caused by hemodynamic changes, either acute volume contraction or rapid upregulation of TGF, rather than by structural damage. We also noticed that SGLT2 inhibitors had a larger eGFR reduction than placebo and a smaller eGFR reduction than active control. However, confounding by trial duration was likely considering that all 9 trials with active control had a duration of 52 weeks or longer. As none of the included studies had a stratification of hyperfiltrative patients and only one study had a mean baseline eGFR in the hyperfiltrative range (Lu et al., 2016), we were unable to evaluate eGFR changes in the subgroup of patients with hyperfiltration, as has been reported before (Cherney et al., 2014).

Regarding albuminuria, we found that SGLT2 inhibition was associated with significant urine ACR reduction in type 2 diabetic patients in CKD, but not in type 2 diabetic patients in general. The lack of a substantial urine ACR reduction in patients without CKD may be explained by their low baseline urine ACR, i.e., the urine ACR in normoalbuminuric patients does not decrease by more than 30 mg/g. Although previous pooled analyses reported positive results of SGLT2 inhibitors in albuminuria reduction, they largely included patients with microalbuminuria and macroalbuminuria at baseline (Cherney et al., 2016; Fioretto et al., 2016; Heerspink et al., 2016; Yamout et al., 2014). Our results, in accord with findings of previous clinical trials (Barnett et al., 2014; Wanner et al., 2016) and post hoc analyses (Cherney et al., 2016; Heerspink et al., 2016; Yamout et al., 2014), demonstrate the role of SGLT2 inhibitors in slowing the progression of albuminuria. However, it is still unclear whether SGLT2 inhibitors can prevent incident albuminuria. Empagliflozin was observed to reduce incident albuminuria in the EMPA-REG RENAL trial, but not in the EMPA-REG OUTCOME trial (Barnett et al., 2014; Wanner et al., 2016). Our analysis did not identify significant subgroup difference between different SGLT2 inhibitors either in eGFR or in urine ACR, suggesting drug class rather than molecule specific effects. However, confirmation from long-term trials conducted in different SGLT2 inhibitors, such as the CREDENCE trial (NCT02065791), is still needed.

Given the important yet incomplete renoprotective roles of RAAS inhibitors in diabetic nephropathy (Bilous et al., 2009; Lewis et al., 1993; Lewis et al., 2001; Mauer et al., 2009), another question to consider is whether SGLT2 inhibition has additive renoprotective effects to RAAS inhibition. SGLT2 inhibition reduces intraglomerular pressure by constriction of afferent arterioles through upregulation of TGF, while RAAS blockage mainly dilates efferent arterioles. Besides acting at different intrarenal sites, SGLT2 inhibition activates RAAS systematically, probably due to volume contraction (Cherney et al., 2014). Thus, it is plausible for SGLT2 inhibition and RAAS inhibition to work synergistically and there is accumulating evidence for this synergy. Weber et al. (2016) reported that in diabetic patients on RAAS inhibitors, addition of dapagliflozin was associated with better blood pressure control, no significant difference in eGFR and a trend toward albuminuria reduction relative to placebo. In the EMPA-REG OUTCOME trial, where 80.7% of patients were taking RAAS inhibitors, empagliflozin was associated with slower progression of nephropathy than placebo (Wanner et al., 2016). Future trials focusing on patients with background RAAS inhibition, such as the CREDENCE study, will further shed light on this issue.

Despite rigorous methodology, our study has several limitations. First, the evaluation of eGFR changes in type 2 diabetic patients overall might be obscured by mixing short-term eGFR decrease and long-term eGFR preservation. Second, substantial heterogeneity in analyses of both eGFR and urine ACR may have complicated the interpretation of our data. Third, our study used surrogate endpoints, including eGFR and urine ACR, rather than hard endpoints, such as progression of nephropathy or renal and cardiovascular mortality (Stevens, Greene & Levey, 2006). The EMPA-REG OUTCOME trial provided solid evidence that empagliflozin reduced the risk of progression of albuminuria, doubling of serum creatinine, initiation of renal replacement therapy and cardiovascular death in type 2 diabetic patients with high cardiovascular risk (Wanner et al., 2016), findings to be confirmed by the ongoing CREDENCE trial with primary renal outcomes.

In conclusion, SGLT2 inhibition was not associated with significant changes in eGFR in type 2 diabetic patients, which may result from a mixture of an initial reduction of eGFR and long-term renal function preservation. SGLT2 inhibition was associated with albuminuria reduction in type 2 diabetic patients with CKD. The therapeutic value of SGLT2 inhibitors in the prevention and management of diabetic nephropathy warrants further study.

##  Supplemental Information

10.7717/peerj.3405/supp-1 Figure S1 Risk of bias summary. Study quality were evaluated using the ‘Risk of bias’ assessment tool from the Cochrane Handbook for Systematic Reviews of Interventions, version 5.1. Green, yellow and red bars represent low, unclear and high risk of bias, respectively.

10.7717/peerj.3405/supp-2 Figure S2 Risk of bias graph Study quality were evaluated using the ‘Risk of bias’ assessment tool from the *Cochrane Handbook for Systematic Reviews of Interventions*, version 5.1. Green, yellow and red dots represent low, unclear and high risk of bias, respectively.

10.7717/peerj.3405/supp-3 Figure S3 Funnel plot for eGFR with Egger’s regression There is no statistically significant publication bias.( *p* = 0.057).

10.7717/peerj.3405/supp-4 Figure S4 Funnel plot for urine albumin/creatinine ratio (ACR) with Egger’s regression There is substantial publication bias (*p* = 0.002).

10.7717/peerj.3405/supp-5 Supplemental Information 1 Raw data

10.7717/peerj.3405/supp-6 Supplemental Information 2 PRISMA checklist

10.7717/peerj.3405/supp-7 Supplemental Information 3 Search strategy

10.7717/peerj.3405/supp-8 Supplemental Information 4 Formula for imputing change from baseline standard deviation using a correlation coefficient

10.7717/peerj.3405/supp-9 Supplemental Information 5 Formulae for group combination

10.7717/peerj.3405/supp-10 Supplemental Information 6 Detailed description of the study selection process

10.7717/peerj.3405/supp-11 Supplemental Information 7 Rationale of the systematic review

## Additional Information and Declarations

### Data Availability

The following information was supplied regarding data availability: The raw data has been supplied as Supplementary File.
