# Supplementary figures and images for: Effects of sodium-glucose co-transporter 2 (SGLT2) inhibition on renal function and albuminuria in patients with type 2 diabetes: a systematic review and meta-analysis
Source: PeerJ. 2017 Jun 27;5:e3405. doi: 10.7717/peerj.3405 (PMC5490461; doi:10.7717/peerj.3405)

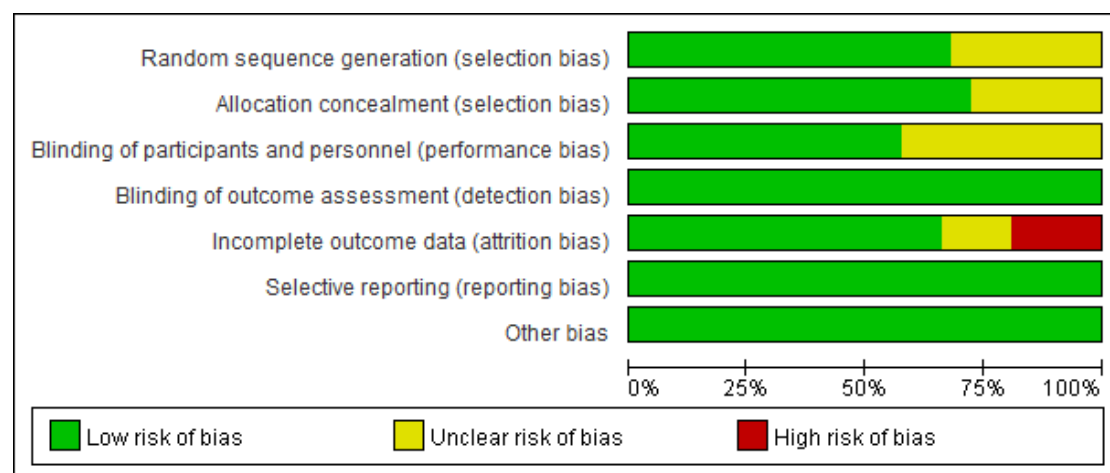

Supplement: Figure S1 — Study quality were evaluated using the ‘Risk of bias’ assessment tool from the Cochrane Handbook for Systematic Reviews of Interventions, version 5.1. Green, yellow and red bars represent low, unclear and high risk of bias, respectively. [file peerj-05-3405-s001.pdf]

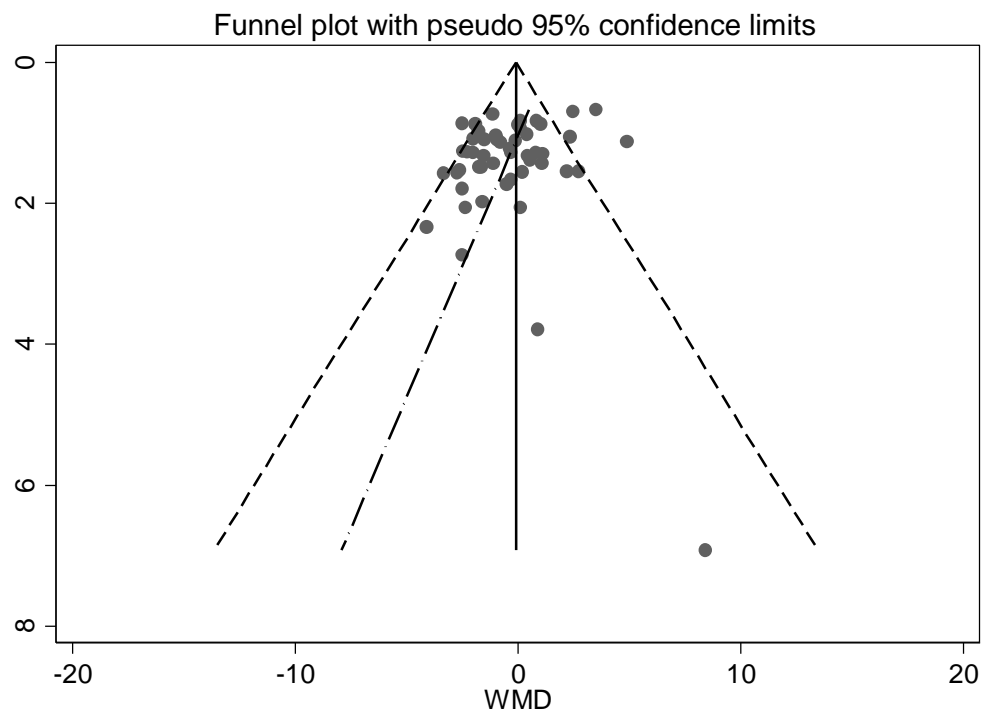

Supplement: Figure S3 — There is no statistically significant publication bias.( p = 0.057). [file peerj-05-3405-s003.pdf]

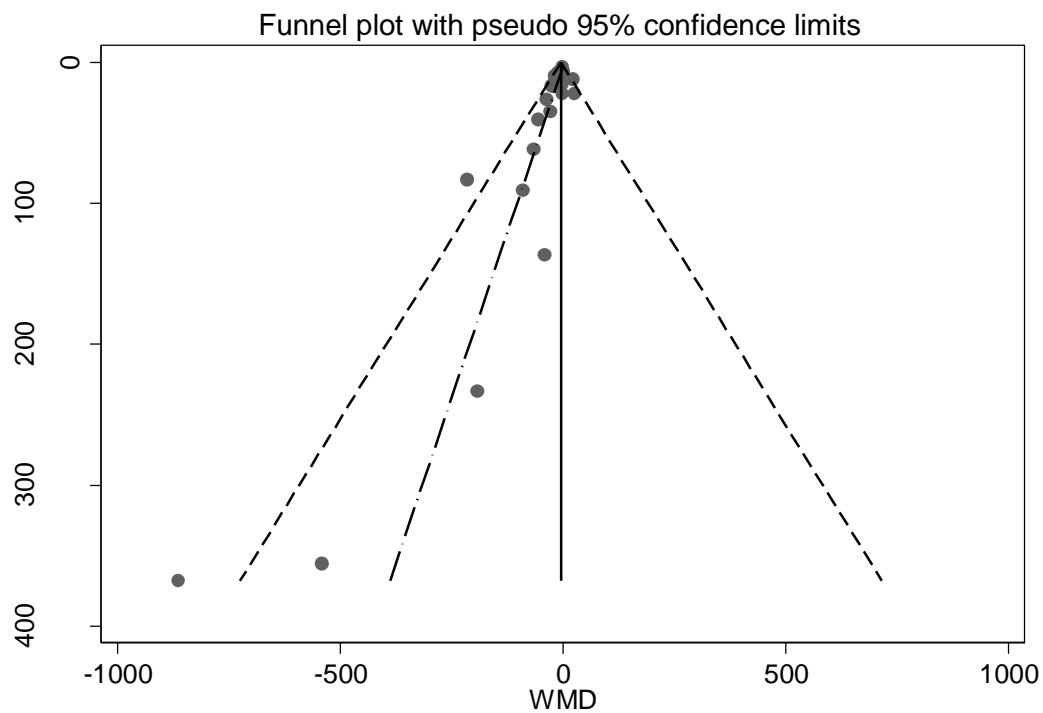

Supplement: Figure S4 — There is substantial publication bias (p = 0.002). [file peerj-05-3405-s004.pdf]
