# Supplementary material for: Effects of sodium-glucose co-transporter 2 (SGLT2) inhibition on renal function and albuminuria in patients with type 2 diabetes: a systematic review and meta-analysis
Source: PeerJ. 2017 Jun 27;5:e3405. doi: 10.7717/peerj.3405 (PMC5490461; doi:10.7717/peerj.3405)
Supplement: Figure S2 — Study quality were evaluated using the ‘Risk of bias’ assessment tool from the Cochrane Handbook for Systematic Reviews of Interventions, version 5.1. Green, yellow and red dots represent low, unclear and high risk of bias, respectively. [file peerj-05-3405-s002.pdf]

	Random sequence generation (selection bias)	Allocation concealment (selection bias)	Blinding of participants and personnel (performance bias)	Blinding of outcome assessment (detection bias)	Incomplete outcome data (attrition bias)	Selective reporting (reporting bias)	Other bias
Bailey 2015	+	+	+	+	+	+	+
Barnett 2014	+	+	+	+	+	+	+
Bode 2013	+	+	+	+	-	+	+
Bolinder 2012	+	+	+	+	+	+	+
Cefalu 2013	+	+	+	+	-	+	+
DeFronzo 2015	?	+	?	+	?	+	+
Fonseca 2013	?	?	?	+	+	+	+
Forst 2014	+	+	+	+	-	+	+
Haring 2013	+	+	?	+	+	+	+
Haring 2014	+	+	?	+	+	+	+
Inagaki 2014	+	+	+	+	?	+	+
Ji 2014	+	+	+	+	+	+	+
Ji 2015	+	?	+	+	+	+	+
Kadowaki 2014	+	+	?	+	?	+	+
Kaku 2014	+	+	+	+	-	+	+
Kashiwagi 2015 DI BRIGHTEN	?	?	?	+	+	+	+
Kashiwagi 2015 DI EMIT	?	+	+	+	+	+	+
Kashiwagi 2015 DI SPOTLIGHT	?	+	+	+	+	+	+
Kashiwagi 2015 DOM LANTERN	?	?	?	+	+	+	+
Kohan 2014	?	?	?	+	-	+	+
Kovacs 2014	+	+	?	+	+	+	+
Lambers Heerspink 2013	+	+	+	+	?	+	+
Lavalle-Gonzalez 2013	+	?	+	+	-	+	+
Lewin 2015	?	+	?	+	?	+	+
Lu 2016	+	+	+	+	+	+	+
Nauck 2011	+	+	+	+	+	+	+
Nishimura 2015	?	?	?	+	?	+	+
Qiu 2014	?	?	+	+	+	+	+
Ridderstrale 2014	+	+	+	+	+	+	+
Rodbard 2016	+	+	?	+	-	+	+
Roden 2013	+	+	+	+	+	+	+
Rosenstock 2014	?	+	?	+	+	+	+
Rosenstock 2015	+	+	?	+	+	+	+
Rosenstock 2016	+	+	+	+	+	+	+
Ross 2015	?	?	?	+	?	+	+
Schernthaner 2013	+	+	+	+	-	+	+
Schumm-Draeger 2015	+	+	+	+	+	+	+
Sha 2014	?	?	?	+	+	+	+
Strojek 2011	+	?	+	+	+	+	+
Tikkanen 2015	+	+	?	+	+	+	+
Wanner 2016	+	+	+	+	+	+	+
Weber 2016	+	+	+	+	+	+	+
Wilding 2009	?	?	?	+	+	+	+
Wilding 2012	+	+	+	+	+	+	+
Wilding 2013 DOM	?	?	?	+	+	+	+
Wilding 2013 IJCP	+	+	+	+	-	+	+
Yale 2013	+	+	?	+	+	+	+
