# Supplementary material for: Effects of sodium-glucose co-transporter 2 (SGLT2) inhibition on renal function and albuminuria in patients with type 2 diabetes: a systematic review and meta-analysis
Source: PeerJ. 2017 Jun 27;5:e3405. doi: 10.7717/peerj.3405 (PMC5490461; doi:10.7717/peerj.3405)
Supplement: Supplemental Information 3 [file peerj-05-3405-s007.docx]

**Item S1 Search strategy**

**Pubmed 2016/6/19 1243 results**

#1 dapagliflozin OR canagliflozin OR empagliflozin OR sergliflozin OR ipragliflozin OR tofgliflozin OR luseogliflozin OR atigliflozin ORbi 44847 OR ertugliflozin OR remogliflozin OR sotagliflozin

#2 dapagliflozin[nm] OR canagliflozin[nm] OR empagliflozin[nm] OR sergliflozin[nm] OR 'sergliflozin etabonate[nm] OR ipragliflozin[nm] OR luseogliflozin[nm] OR remogliflozin etabonate[nm]

#3 SGLT2 inhibitor OR SGLT2-inhibitor OR sodium glucose cotransporter 2 inhibitor OR sodium-glucose cotransporter 2 inhibitor OR sodium/glucose cotransporter 2 inhibitor OR sodium glucose co-transporter 2 inhibitor OR sodium-glucose co-transporter 2 inhibitor OR sodium-dependent glucose transporter 2 inhibitor

#4 ( "Sodium-Glucose Transporter 2/administration and dosage"[mh] OR "Sodium-Glucose Transporter 2/adverse effects"[mh] OR "Sodium-Glucose Transporter 2/analysis"[mh] OR "Sodium-Glucose Transporter 2/antagonists and inhibitors"[mh] OR "Sodium-Glucose Transporter 2/blood"[mh] OR "Sodium-Glucose Transporter 2/drug effects"[mh] OR "Sodium-Glucose Transporter 2/metabolism"[mh] OR "Sodium-Glucose Transporter 2/pharmacokinetics"[mh] OR "Sodium-Glucose Transporter 2/pharmacology"[mh] OR "Sodium-Glucose Transporter 2/physiology"[mh] OR "Sodium-Glucose Transporter 2/therapeutic use"[mh] ) AND inhibitor

#5 #1 OR #2 OR #3 OR #4

**Embase2016/6/19 2929 results**

#1 ‘sodium glucose cotransporter 2 inhibitor’/exp

#2 atigliflozin OR ‘bi 44847’ OR canagliflozin OR dapagliflozin OR empagliflozin OR ertugliflozin OR ipragliflozin OR luseogliflozin OR remogliflozin OR sergliflozin OR sotagliflozin OR tofogliflozin

#3 'sglt2 inhibitor' OR 'sglt2-inhibitor' OR 'sodium glucose cotransporter 2 inhibitor' OR 'sodium-glucose cotransporter 2 inhibitor' OR 'sodium/glucose cotransporter 2 inhibitor' OR 'sodium glucose co-transporter 2 inhibitor' OR 'sodium-glucose co-transporter 2 inhibitor' OR 'sodium-dependent glucose transporter 2 inhibitor'

#4 #1 OR #2 OR #3

**Cochrane Central Register of ControlledTrials (CENTRAL) 2016/6/19 533 results**

#1 dapagliflozin OR canagliflozin OR empagliflozin OR sergliflozin OR ipragliflozin OR tofgliflozin OR luseogliflozin OR atigliflozin ORbi 44847 OR ertugliflozin OR remogliflozin OR sotagliflozin [search all text, word variations have been searched]

#2 SGLT2 inhibitor OR SGLT2-inhibitor OR sodium glucose cotransporter 2 inhibitor OR sodium-glucose cotransporter 2 inhibitor OR “sodium/glucose cotransporter 2 inhibitor” OR sodium glucose co-transporter 2 inhibitor OR sodium-glucose co-transporter 2 inhibitor OR sodium-dependent glucose transporter 2 inhibitor [search all text, word variations have been searched]

#3 [mh “Sodium-Glucose Transporter 2”] with explosion

#4 inhibitor [search all text, word variations have been searched]

#5 #3 AND #4

#6 #1 OR #2 OR #5 [Trials]
