# Supplementary material for: Effects of sodium-glucose co-transporter 2 (SGLT2) inhibition on renal function and albuminuria in patients with type 2 diabetes: a systematic review and meta-analysis
Source: PeerJ. 2017 Jun 27;5:e3405. doi: 10.7717/peerj.3405 (PMC5490461; doi:10.7717/peerj.3405)
Supplement: Supplemental Information 4 [file peerj-05-3405-s008.docx]

**Item S2 Formula for imputing change-from-baseline standard deviation using a correlation coefficient**

For studies where none of standard deviation, standard error or 95% confidential interval of change-from-baseline was reported, standard deviation of change-from-baseline was imputed using the formula below as recommended in Chapter 16.1.3.2 of the *Cochrane Handbook for Systematic Reviews of Interventions*, version 5.1. Corr stands for correlation coefficient, which was set at 0.8.

$${SD}_{change}=\sqrt{{{SD}_{baseline}}^{2}+{{SD}_{final}}^{2}-2\times corr\times{SD}_{baseline}\times{SD}_{final}}$$
