# Supplementary material for: Effects of sodium-glucose co-transporter 2 (SGLT2) inhibition on renal function and albuminuria in patients with type 2 diabetes: a systematic review and meta-analysis
Source: PeerJ. 2017 Jun 27;5:e3405. doi: 10.7717/peerj.3405 (PMC5490461; doi:10.7717/peerj.3405)
Supplement: Supplemental Information 5 [file peerj-05-3405-s009.docx]

**Item S3 Formulae for group combination**

For studies with more than one SGLT2 treatment arms, groups were combined to create a single treatment arm using the following formulae as recommended in Chapter 7.7.3.8 of the *Cochrane Handbook for Systematic Reviews of Interventions*, version 5.1.

|  | Group 1 | Group 2 | Combined groups |
| --- | --- | --- | --- |
| Sample size | N1 | N2 | N1+N2 |
| Mean | M1 | M2 | $\frac{N1*M1+N2*M2}{N1+N2}$ |
| SD | SD1 | SD2 | $\sqrt{\frac{\left( N1-1 \right)*{SD1}^{2}+\left( N2-1 \right)*{SD2}^{2}+\frac{N1*N2}{N1+N2}(M1^{2}+{M2}^{2}-2*M1*M2)}{N1+N2-1}}$ |
