# Supplementary material for: Effects of sodium-glucose co-transporter 2 (SGLT2) inhibition on renal function and albuminuria in patients with type 2 diabetes: a systematic review and meta-analysis
Source: PeerJ. 2017 Jun 27;5:e3405. doi: 10.7717/peerj.3405 (PMC5490461; doi:10.7717/peerj.3405)
Supplement: Supplemental Information 6 [file peerj-05-3405-s010.docx]

**Details of study selection and characteristics**

The results of the literature search and study selection are shown in Figure 1. In total, 4,705 publications were identified in the electronic search, of which 777 were discarded as duplicates and 3,209 were discarded due to lack of relevance to the study question. Of the 719 studies retrieved for further evaluation, 671 were removed for the following reasons: no published manuscript (n=447), not an RCT in type 2 diabetic patients (n=123), multiple publications from the same study (n=25), no report of renal outcome (n=55) and renal data not extractable(n=22). The search process led to inclusion of 47 studies (Bailey et al. 2015; Barnett et al. 2014; Bode et al. 2013; Bolinder et al. 2012; Cefalu et al. 2013; DeFronzo et al. 2015; Fonseca et al. 2013; Forst et al. 2014; Haring et al. 2014; Haring et al. 2013; Inagaki et al. 2014; Ji et al. 2015; Ji et al. 2014; Kadowaki et al. 2014; Kaku et al. 2014; Kashiwagi et al. 2015a; Kashiwagi et al. 2015b; Kashiwagi et al. 2015c; Kashiwagi et al. 2015d; Kohan et al. 2014; Kovacs et al. 2014; Lambers Heerspink et al. 2013; Lavalle-Gonzalez et al. 2013; Lewin et al. 2015; Lu et al. 2016; Nauck et al. 2011; Nishimura et al. 2015; Pieber et al. 2015; Qiu et al. 2014; Ridderstrale et al. 2014; Rodbard et al. 2016; Roden et al. 2013; Rosenstock et al. 2016; Rosenstock et al. 2014; Rosenstock et al. 2015; Ross et al. 2015; Schernthaner et al. 2013; Schumm-Draeger et al. 2015; Sha et al. 2014; Strojek et al. 2011; Tikkanen et al. 2015; Wanner et al. 2016; Weber et al. 2016; Wilding et al. 2013a; Wilding et al. 2013b; Wilding et al. 2009; Wilding et al. 2012; Yale et al. 2013) with 22,843 participants in our meta-analysis.

The characteristics of the included studies are shown in Table 1. Of the 47 studies, 46 studies with 22,603 participants reported changes in eGFR, and 17 studies with 7,285 participants reported changes in urine ACR. Five SGLT2 inhibitors, including dapagliflozin, canagliflozin, empagliflozin, ipragliflozin and tofogliflozin, were assessed. A total of 38 studies were placebo-controlled, and 9 were controlled by other antidiabetic medications, including metformin, glimepiride, glipizide, linagliptin, and sitagliptin. The trial durations ranged from 4 weeks to 156 weeks. Six studies reported outcomes of CKD subjects. In other studies, the mean baseline eGFR ranged from 76.7 to 149.2 ml/min per 1.73 m2, and the mean baseline urine ACR ranged from 6.7 to 143.7 mg/g. None of the studies reported outcomes in a group of patients with hyperfiltration. Collection of data on concomitant use of RAAS inhibitors was planned a priori; however, this was impeded as only 6 studies reported the number of patients using RAAS inhibitors (Barnett et al. 2014; Bode et al. 2013; Lambers Heerspink et al. 2013; Sha et al. 2014; Wanner et al. 2016; Weber et al. 2016), among which only one study reported outcomes with stratification according to RAAS inhibitor usage (Weber et al. 2016).

Quality assessment (Figures S1 and S2) revealed that 32 studies described random sequence generation, and that 34 described allocation concealment. Twenty-seven studies described blinding of participants and personnel. All 47 studies had a low risk of detection or reporting bias, and 31 studies had a low risk of attrition bias.
