# Supplementary material for: Effects of sodium-glucose co-transporter 2 (SGLT2) inhibition on renal function and albuminuria in patients with type 2 diabetes: a systematic review and meta-analysis
Source: PeerJ. 2017 Jun 27;5:e3405. doi: 10.7717/peerj.3405 (PMC5490461; doi:10.7717/peerj.3405)
Supplement: Supplemental Information 7 [file peerj-05-3405-s011.docx]

**1. The rationale for conducting the meta-analysis;**

1) Sodium-glucose co-transporter 2 (SGLT2) inhibitors are gaining popularity due to various beneficial effects in addition to glycemic control, including BP lowering, weight control and reduction in cardiovascular mortality in type 2 diabetic patients with high cardiovascular risk.

2) SGLT2 inhibition has profound effects on renal hemodynamics. By blocking glucose and sodium reabsorption at the proximal tubule, SGLT2 inhibitors increase sodium delivery to the macula densa, and upregulate tubuloglomerular feedback (TGF). Thus, it is postulated that SGLT2 inhibition alleviates glomerular hyperfiltration, reduces albuminuria and potentially slows the progression of diabetic nephropathy.

**2. The contribution that the meta-analysis makes to knowledge in light of previously published related reports, including other meta-analyses and systematic reviews.**

A number of clinical trials have reported kidney-related outcomes after SGLT2 inhibitor use. This systematic review and meta-analysis summarizes available evidence to thoroughly characterize the effects of SGLT2 inhibition on eGFR and albuminuria in diabetic patients. A previous meta-analysis (Liu et al. 2015) reported the effects of SGLT2 inhibitors on eGFR. However, Liu et al. 2015 only included trials with a duration between 52 weeks and 104 weeks before December 2014. Our analysis is more updated, includes trials of different durations, and also includes albuminuria as an outcome.

In our analysis, we noted an intitial eGFR reduction and long-term eGFR preservation after SGLT2 inhibitor use. The overall change in eGFR was not significant in diabetic patients with or without CKD. SGLT2 inhibition was associated with a trend toward albuminuria reduction, which was statistically significant in diabetic patients with CKD.
